# Diagnostic Strategy for Suspected Pulmonary Embolism in Emergency Departments Based on the 4-Level Pulmonary Embolism Clinical Probability Score: Study Protocol of SPEED&PEPS Trial

**DOI:** 10.3390/diagnostics12123101

**Published:** 2022-12-09

**Authors:** Pierre-Marie Roy, Thomas Moumneh, Andrea Penaloza, Jeannot Schmidt, Sandrine Charpentier, Luc-Marie Joly, Jérémie Riou, Delphine Douillet

**Affiliations:** 1 Angers University Hospital, Emergency Department, Univ Angers, MitoVasc UMR CNRS 6015—INSERM 1083, FCRIN, INNOVTE, 49000 Angers, France; 2 Cliniques Universitaires St-Luc, Emergency Department, Université Catholique de Louvain, 1200 Bruxelles, Belgium; 3 Clermont-Ferrand University Hospital—Gabriel Montpied, Emergency Department, FCRIN, INNOVTE, 63000 Clermont-Ferrand, France; 4 Toulouse University Hospital, Emergency Department, 31300 Toulouse, France; 5 Rouen University Hospital, Emergency Department, 76031 Rouen, France; 6 Angers University Hospital, Methodology and Biostatistics Department, Delegation to Clinical Research and Innovation, Univ Angers, MINT UMR INSERM 1066—CNRS 6021, 49000 Angers, France

**Keywords:** pulmonary embolism, over-testing, study protocol, diagnostic strategy, clinical probability, cluster-randomized trial, pragmatic trial

## Abstract

*Introduction:* Several strategies have been devised to safely limit the use of thoracic imaging in patients suspected of pulmonary embolism (PE). However, they are based on different rules for clinical probability (CP) assessment, rendering their combination difficult. The four-level pulmonary embolism probability score (4PEPS) allows the combination of all other strategies using a single CP assessment. *Methods and analysis:* Pragmatic cluster-randomized trial in 20 EDs. Patients with suspected PE will be included and followed for 90 days (number of patients to be included: 2560, 1280 in each arm). Ten centers will be allocated to the control group where physicians will be free to do as they see fit but they will be given the recommendation to apply a validated strategy. Ten centers will be allocated to the interventional group where the physicians will be given the recommendation to apply the 4PEPS strategy. The primary objective will be to demonstrate that the application of the 4PEPS strategy by the emergency physicians, in comparison to current practices, (i) does not increase the risk of serious events related to diagnostic strategies and (ii) significantly reduces the use of thoracic imaging. *Ethics and dissemination:* The study will be submitted for approval to an institutional ethics review board for all participating centers. If successful, the SPEED&PEPS trial will have an important impact for patients suspected of PE limiting their irradiation and for public health in substantial savings in terms of the direct cost of diagnostic investigations and the indirect cost of hospitalizations due to waiting times or delayed harmful effects. *Funding:* This work is funded by a French Public Health grant (PREPS-N 2019). The funding source plays no role in the study design, data collection, analysis, interpretation or the writing of the manuscript. *Trial registration:* ongoing. *Trial status:* not started.

## 1. Introduction

The increased use of X-ray examinations in diagnostic approaches used at emergency departments is a major public healthcare issue in three ways: (i) for patients, due to the risks linked to cumulative radiation exposure and the resulting longer care time; (ii) for healthcare professionals, because it contributes to overcrowding in emergency departments; and (iii) for society as a whole due to the cost [1]. Suspected pulmonary embolism (PE) is without doubt one of the most striking examples [2]. Given that PE is a potentially life-threatening condition without any specific symptoms or clinical signs, it is often put forward as a possible diagnosis, which leads to ancillary investigations—mainly a D-dimer test and/or computed tomography pulmonary angiography (CTPA) [3,4,5]. In the last twenty years, the number of patients who undergo a CTPA to screen for PE has multiplied by ten while the PE prevalence among investigated patients has dropped from more than 25% to less than 5% [2]. At the same time, the incidence of PE increased but without significant impact on morbidity and mortality [6]. Indeed, PE-related mortality continues to decrease but to a lesser extent than before the wide spread of CTPA and to a much lesser extent than the rise in PE diagnosis [6]. This observation has highlighted another risk linked to the overuse of CTPA, namely overdiagnosis. The greater the use of CTPA, the greater the risk of false positives, especially when the quality of the CTPA is suboptimal. This is combined with the advances in CTPA’s abilities to show subsegmental PE which may not be of clinical relevance and lead to initiating unnecessary anticoagulant treatment [7].

Several strategies have therefore been devised to limit the use of CTPA, based on the formalized assessment of clinical probability (CP) and/or by modifying the cut-off value for D-dimer tests. Yet, each of these strategies is based on a different and specific method of assessing CP: the PERC (pulmonary embolism rule-out criteria) and implicit assessment for ruling out PE on clinical criteria [8,9,10], the YEARS rule or the adapted Wells score for ruling out PE on CP-adjusted D-dimer cut-off [11,12], and the revised Geneva score or the Wells score for ruling out PE on age-adjusted D-dimer cut-off [13,14]. This makes them difficult to use and combine, and can lead to non-expert physicians misusing these rules or relying solely on their empirical assessment, thereby exposing themselves to the risk of a misdiagnosis (loss of reliability) or inappropriate use of CT (loss of efficacy). 

We have developed and validated a score that combines all these possibilities into a single rule: 4PEPS (four-level pulmonary embolism probability score) [15]. Based on ISTH recommendations and depending on the prevalence of VTE in the population, the acceptable number of false negatives has been set to 2% [16]. Using 12 variables, 4PEPS defines four levels of CP that rule out a PE, namely (1) based only on clinical data (very low CP: PEPS < 0), (2) based on a D-dimer level < 1000 µg/L (low CP: PEPS ≥ 0 et < 5), (3) based on a D-dimer level with an age-adjusted cut-off value (moderate CP: PEPS ≥ 5 and <12), or (4) the diagnosis cannot reliably be ruled out based on a D-dimer test (high CP: PEPS ≥ 12) (Table 1). Assessed retrospectively based on large databases, the 4PEPS strategy seems to be more efficient and just as safe as the other strategies [15,17,18]. 

Our research hypothesis is that a diagnostic strategy based on 4PEPS will help reduce the use of thoracic imaging without increasing the risk of serious adverse events linked to the diagnostic approach. 

## 2. Study Design

We will test this study hypothesis in a prospective pragmatic cluster-randomized trial, with half of the centers in a control group where physicians will be free to do as they see fit but they will be given the recommendation to apply a validated strategy, and half of the centers will be in the interventional group where the physicians will be given the recommendation to apply the 4PEPS strategy [19,20]. 

## 3. Study Objectives and Endpoints

### 3.1. Primary Objective and Endpoints 

The primary objective is to demonstrate that the application of the 4PEPS strategy by the emergency physicians in comparison to current practices (i) does not increase the risk of serious events related to diagnostic strategies and (ii) significantly reduces the use of thoracic imaging. 

The primary safety endpoint will be the rate of serious events in the 90 days following inclusion:

- Serious adverse events related to diagnostic testing (leading to hospitalization or prolongation of hospitalization, permanent inability or incapacity, and death).
- Symptomatic thromboembolic events in patients not diagnosed with PE in the emergency department or new thromboembolic events in patients diagnosed with PE.
- Death related to PE (initial or recurrent PE).
- Major bleeding related to the anticoagulant treatment prescribed for pulmonary embolism according to ISTH criteria [21].

All events must be symptomatic, documented, and confirmed by the clinical adjudication committee blinded to the study group. For analysis of deaths in cases where an autopsy is not performed, a precise definition of the elements indicating that the death is possibly related to pulmonary embolism will be used according to recent ISTH proposals [22].

The primary efficacy endpoint will be the rate of thoracic imaging among all included patients with suspected PE. A relative reduction of at least 20% is expected.

The following thoracic examinations will be considered if they are performed at the request of the emergency physician in search of PE and this during the passage in the emergency department and/or within 72 h of inclusion:

- CTPA.
- Planar perfusion or perfusion–ventilation pulmonary scintigraphy.
- SPECT scintigraphy.

The examinations have to be mentioned in the patient’s record and be the subject of a report, with analysis of the file taken as proof of this. 

### 3.2. Secondary Objective(s) and Endpoint(s)

The first secondary objective is to demonstrate that the risk of false-negative diagnosis with the 4PEPS strategy is very low in accordance with the ISTH criteria for validation of rule-out PE diagnostic strategies. The endpoint will be the false-negative rate defined as the rate of thromboembolic events occurring in the 90 days following inclusion in patients for whom PE was ruled out according to the 4PEPS strategy and who were not treated with anticoagulants. ISTH recommendations state the upper limit of the 95% confidence interval (95%CI) of this 90-day thromboembolic event rate should be less than 1.82 + 0.00528* prevalence, i.e., a rate ≤1.89% for a prevalence of 14% [16].

The second secondary objective is to demonstrate that the 4PEPS strategy (intervention group) is not inferior to current practices (control group) as regards the risk of false-negative diagnosis. The false-negative rate will be defined as the rate of thromboembolic events occurring in the 90 days following inclusion in patients for whom PE was ruled out and who were not treated with anticoagulants. 

The other secondary objectives are to evaluate and compare to usual practice the impact of the 4PEPS strategy as regards the rate of D-dimer measurement and the length of time spent in the emergency department by patients with suspected PE.

The ancillary objectives are to assess the rate of incidental findings on CTPA, the acceptance by physicians and their use in everyday practice of the clinical decision support tool SPEED [23], as regards the rate of patients for whom the SPEED application is used in real time (i.e., when the data that enable the automatic calculation of the clinical probability and the reminder of the appropriate diagnostic strategy have been entered before any additional examinations are performed, in particular, the D-dimer test); and to evaluate the appropriateness of diagnostic management according to international guidelines and if it differs when the SPEED application is used in real time or not. 

We will also evaluate and compare to usual practice the medico-economic impact of the 4PEPS strategy in terms of cost per serious clinical event related to the diagnostic procedure avoided in the 90 days following inclusion (cost–efficacy analysis). The endpoint will be the differential cost–efficacy ratio at 90 days.

## 4. Study Settings and Population

### 4.1. Centers

Twenty centers will be selected based on their participation in previous trials on PE diagnosis in emergency departments and their possibility of recruitment.

Inclusion criteria for study participants

- Admission to an emergency department participating in the study.
- Suspected PE due to thoracic symptoms (dyspnea, chest pain, or hemoptysis) and/or syncope without any other obvious explanation after clinical examination and possible additional first-line tests (ECG, chest X-ray, or routine lab work-up not including D-dimer test).
- Free, prior, and informed consent to participate in the study.

### 4.2. Non-Inclusion Criteria for Study Participants

- Age < 18 years.
- Known result of a specific diagnostic examination for PE (D-dimer test, thoracic CT angiography, pulmonary scintigraphy, or venous ultrasound of the lower limbs).
- Hemodynamic instability (systolic blood pressure < 90 mmHg or more than 40 mmHg lower than usual for more than 15 min).
- Curative dose of anticoagulant in place for more than two days prior to inclusion.
- 3-month follow-up not possible.
- Pregnant or parturient patient.
- Patient in detention by judicial or administrative decision.
- Patient undergoing compulsory psychiatric treatment.
- Patient placed under a legal protection measure.
- Patient incapable of giving free and informed consent.

## 5. Experimental Plan and Randomization

SPEED&PEPS will be an open-labelled cluster-randomized study, with 10 centers being allocated to the control group and 10 centers to the interventional group. Before randomization, participating centers will perform a preliminary assessment of the number of patients being investigated for suspected PE per month and of the rate of imaging test performed in patients suspected of PE in order to have an accurate idea of their baseline status [20]. These two criteria will be applied as stratification criteria during randomization to assure comparability between groups. 

SPEED&PEPS will be a pragmatic trial as confirmed by the PRECIS-2 rating of 42 out of 45 on the scale, with 0 corresponding to a fully explanatory study and 45 to a fully pragmatic study (cf. supplemental material) [24]. 

## 6. Description of Study Intervention

### 6.1. Comparison Strategy: Control Group

The investigating physicians of the participating centers in the control group will be free to provide care as they see fit. However, a reminder of national and European guidelines for PE management will be given to them and they will have the recommendation to apply a validated strategy [3,4,5]. To make it easier, the different scores will be included in the clinical help–decision support software called SPEED [23]. Investigators will be asked to enter data about the included patients directly into SPEED, which will act as the study’s electronic case report form (eCRF). A paper version of the CRF will also be available. 

### 6.2. Strategy under Study: Interventional Group

Physicians of the participating centers in the intervention group will have the recommendation to apply the 4PEPS strategy. To make it easier to apply, the 4PEPS score will be included in SPEED [23]. Investigators will be asked to enter the information relating to patients included in the study directly into SPEED before performing any testing. Entering these data will enable the 4PEPS score to be calculated automatically and specific recommendations to be provided. A paper version of the CRF will also be available.

### 6.3. Associated Care and Treatment 

For the duration of the study, physicians will be free to treat and refer patients with a diagnosis of PE. However, they will be reminded of practice guidelines on VTE treatment [3,4,5]. These recommendations will also be accessible via the SPEED clinical decision support tool.

## 7. Study Participant Follow-Up 

Data relating to care at the emergency department and the eventual subsequent hospitalization (characteristics, comorbidities, clinical and paraclinical data, confirmed diagnosis or diagnoses, possible adverse events, and treatment) will be compiled and entered into the eCRF. At discharge from the hospital, patients will be instructed to return to the ED or to contact their family physician in the event of any symptoms that may suggest a possible thromboembolic event or bleeding.

Patients and/or their relatives and family physician(s) will be contacted by phone and/or post or email at the end of the 3-month follow-up period. Using a standardized questionnaire, they will be asked about whether a thromboembolic event or bleeding has occurred; what examinations, consultations, or hospitalizations have taken place; and whether anticoagulant treatment has been introduced. Examination results (biological tests including D-dimer, ultrasonography, thoracic imaging…), hospital discharge summaries, and circumstances and causes of death, if any, will be collected. For each possible event during the follow-up, an adjudication file will be constituted, including reports of all relevant exams. All possible events will be adjudicated by the independent committee, blinded of randomization arm (control or interventional group). 

## 8. Medico-Economic Analysis

The medico-economic analysis will be performed following the recommendations of the French National Authority for Health (HAS) (https://www.has-sante.fr/portail/jcms/r_1499251/fr (accessed on 1 November 2022)) and the CHEERS (Consolidated Health Economic Evaluation Reporting Standards) statement [25]. A cost–efficacy analysis will be performed, aimed at comparing the efficacy of applying the 4PEPS strategy against usual practices in terms of cost for each serious event avoided, from the perspective of health insurance. The timescale will be 90 days corresponding to the evaluation period of the efficacy endpoint.

The result will be shown as a differential cost–efficacy ratio and expressed as a cost per serious event avoided within 90 days (serious adverse event of PE testing, PE-related death, thromboembolic event, or major bleeding). This evaluation will be performed at D90 via a telephone call using a standardized questionnaire.

The evaluation of costs will concern the use of hospital and outpatient treatments and medical equipment (medications and medical devices). 

## 9. Statistical Analysis

### 9.1. General Considerations 

Population characteristics will be described using their mean/standard deviation, median/interquartile range, or percent/95% confidence interval, as appropriate. Univariate comparisons will be made using Student’s, Mann–Whitney, or Fisher’s exact tests, as appropriate. The alpha risk will be set at 5%. All tests will be one-sided. Given that some proportions are expected to be small, confidence intervals will be calculated using the Clopper and Pearson exact method. 

The analysis will be performed using R software (R Foundation for Statistical Computing; 2008. http://www.R-project.org (accessed on 1 November 2022)) and its PropCIs and MASS add-ons (http://CRAN.R-project.org/package=PropCIs (accessed on 1 November 2022)). All objectives will be analyzed on the overall included population (intention-to-treat). A complementary per-protocol analysis will be performed, especially for the 4PEPS strategy in the interventional group. 

### 9.2. Primary Statistical Analysis

The two main endpoints will be analyzed in a hierarchical manner. Firstly, non-inferiority will be analyzed on the safety endpoint with a non-inferiority limit set at 1.5%. If and only if confirmed, superiority analysis on the efficacy endpoint will be performed. Analyses will be performed using a mixed-effects logistic regression in line with recommendations for this type of study [26]. Missing data will be analyzed in order to determine whether they are informative and whether they are likely to cause a potential screening or information bias. 

### 9.3. Secondary Statistical Analysis

The confidence interval of the false-negative rate with the 4PEPS strategy will be calculated on an intention-to-treat basis and then on a per-protocol basis, and its upper limit will be compared with the limit considered acceptable by the ISTH recommendations based on the prevalence in the study: 1.82 + 0.00528* prevalence, i.e., for a prevalence of 14%, an upper limit ≤1.89% [16]. 

Hierarchical analysis of the other secondary outcomes will be conducted using a mixed effects logistic regression. If the primary safety endpoint and then the primary efficacy endpoint are confirmed, the false-negative rate with the 4PEPS strategy will be compared to that of the usual approaches in the control group using a mixed-effects logistic regression with a non-inferiority limit set at 1.35% [27]. If this endpoint is confirmed, then the two other secondary endpoints will be analyzed using the Holm procedure for multiple testing correction.

### 9.4. Medico-Economic Statistical Analysis

The endpoint of the medico-economic analysis will be the cost per serious clinical event avoided within 90 days and will be expressed as the differential cost-efficacy ratio: DCRR = Δ Costs/Δ Efficacy.

Statistical analysis specific to the medico-economic section will be carried out based on a decision tree. 

## 10. Justification of the Number of People to Be Included in the Study

The number of patients to be included will be set to demonstrate in the intervention group versus the control group: (1) non-inferiority on the primary safety endpoint (the rate of patients during follow-up with a fatal PE, thromboembolic event, or major bleeding due to anticoagulant treatment) and (2) superiority on the primary efficacy endpoint (the usage rate of thoracic imaging). 

### 10.1. Safety Endpoint

Considering the participation of 20 centers, 10 centers in each group, an alpha risk of 5%, a power of 80%, a clinical event rate of 1.5% at 3 months in both groups, a non-inferiority limit of 1.5%, an intra-center correlation coefficient of 0.002, and a rate of patients lost to follow-up or non-analyzable of 5%, it will be necessary to enroll 2560 patients, 1280 in each arm.

The serious clinical event rate at 3 months will be estimated by taking into account a risk of adverse event related to diagnostic testing of 0.1%; a PE prevalence of 14% [10,11]; a risk of thromboembolic events in patients for whom the diagnosis is ruled out estimated at 0.8% [15] and in patients for whom the diagnosis is made (14% of the population); a PE-related death rate of 2% [28,29]; major bleeding of 2% [28]; and thromboembolic recurrence of 2% [29]. The intra-center correlation rate at 0.002 and the non-inferiority limit at 1.5% will be estimated on the basis of a recent French study [30].

### 10.2. Efficacy Endpoint 

Considering the participation of 20 centers, an alpha risk of 5%, a power of 80%, an intra-center correlation coefficient of 0.05, and a usage rate of thoracic imaging of 45% in the control group, it will be necessary to include 2128 patients to demonstrate a relative decrease (1-sided analysis) greater than or equal to 20% (an absolute decrease of 9%) between the control and interventional groups in the usage rate of thoracic imaging. 

The intra-center correlation coefficient will be set at 0.05, given that the use of imaging is strongly linked to professional practices, which themselves vary significantly between centers. We estimate that it will be 45% in the control group and 36% in the interventional group on the basis of a previous retrospective assessment [15].

This means the figures previously calculated (2560 patients) will be sufficient for the hierarchical analysis of this endpoint. Nevertheless, an analysis of the usage rate of thoracic imaging in the control group will be performed after the inclusion of 1500 patients, i.e., 750 patients in the control group. If this rate is lower than expected (<45%), a reassessment of the sample size will be performed in order to assure a power of 80% to demonstrate a relative decrease of at least 20% between the control and the interventional groups on the usage rate of thoracic imaging.

## 11. Funding, Ethical Considerations, and Regulations

This work will be funded by a French Public Health grant (PREPS-N 2019) and will be sponsored by the University Hospital of Angers. The funding source and the sponsor will play no role in the study design, data collection, analysis, interpretation, or the writing of the manuscript.

The study file (including the protocol, the summary, the list of associated researchers and the study information letter) will be submitted for approval to the ethics committee (CPP). The study will be only initiated after a favorable opinion for conducting this research.

Informed consent will be obtained for all patients and an information letter will be provided to all participants. The data relating to this study will be processed in accordance with the EU General Data Protection Regulation and the study sponsor will sign an agreement to comply with the “Reference Methodology” (declaration number: 1174822). 

The results of the study will be published in peer-reviewed journals and will be presented at conferences.

## 12. Discussion 

The 4PEPS score was specifically derived to reduce over-testing for PE suspicion using a Bayesian approach. The 4PEPS strategy compared well retrospectively with other strategies but required a formal validation in a prospective trial. SPEED&PEPS will provide this unmet need with a very high level of evidence. Analysis of the rate of thromboembolic events occurring in the 3 months of follow-up in patients considered free of PE and not treated with anticoagulant therapy is the most used method for assessing the diagnostic reliability of a diagnostic exclusion strategy for suspected PE, reflecting the false-negative rate of that strategy [8,9,10,11,12,13,14]. The false-negative rate and its 95% CI are compared to a predetermined cut-off, ideally, that defined according to ISTH recommendations [16]. In SPEED&PEPS, this analysis will be carried out but at a complementary element of the analyses of the overall net clinical benefit and efficacy of the 4PEPS strategy as compared with usual practices.

To assess the effects of an intervention on practice, cluster-randomized controlled studies are considered the most relevant, but they require particular analysis [19,20,31]. In such studies, randomization is carried out by the center, but efficacy is judged at the patient level, which increases the number of subjects to include in order to demonstrate an effect and determines the type of statistical tests to be used [26,32,33,34]. Randomizing patients individually would entail a risk of contaminating the strategies between them [35]. The pragmatic design of SPEED&PEPS will help reconcile healthcare-related organizational constraints and the demands of a rigorous scientific evaluation, and it will be particularly tailored to emergency departments [23,27,30]. To ensure that patients are managed according to good medical practice throughout the study, the physicians will be reminded of relevant recommendations and will have the possibility to use the clinical help–decision support software SPEED. SPEED has been shown to improve the diagnostic management of suspected PE in a prospective randomized multi-center study that compared SPEED to the wide distribution of recommendations in paper form [23].

As part of a de-escalation approach, aimed at limiting the overuse of diagnostic testing, analysis of the efficacy on high-risk and/or costly diagnostic investigations (in this case, CTPA or scintigraphy) must be combined with a safety analysis to ensure that the reduction in investigations is not linked to an increase in the number of serious clinical events related to the diagnostic approach. By drawing on Bayesian principles, the diagnostic approach seeks to make a diagnostic and therapeutic decision that either: (1) makes the diagnosis and initiates anticoagulant treatment when the risks associated with the treatment—mainly serious hemorrhages—are lower than the benefits it may provide on the progression of the disease, mainly in terms of mortality and thromboembolic recurrence; or (2) does not make a diagnosis and does not initiate anticoagulant therapy when the risk of progressive thromboembolic pathology, still in terms of mortality or thromboembolic recurrence, is less than the risk of therapeutic side effects [36]. Thus, in addition to severe adverse events directly related to investigations, the progression of PE to death or the occurrence of a new thromboembolic event (therapeutic defect or failure) and the occurrence of a serious hemorrhage (side effect of anticoagulant therapy) are serious clinical events related to the diagnostic strategy. The overall rate of serious clinical events occurring during follow-up reflects the net clinical benefit of the diagnostic approach and the resulting therapeutic decision. It is assessed on all included patients with suspected PE and can be used as a basis for medico-economic analysis.

The hierarchical analysis applied in SPEED&PEPS will allow us to address the questions of efficacy and safety without increasing the alpha risk through multiple tests. If our research hypothesis is confirmed, this will have a major impact for patients and for public health. For patients, the reduction in the use of imaging tests will make it possible to limit the cumulative radiation exposure, the impact of which is now known in terms of the risk of radiation-induced cancer, in particular breast cancer, for women. It will reduce the time it takes to manage patients with suspected PE in the emergency department, as patients that undergo thoracic imaging take an average of 4 h longer to treat than those who do not require it. Our study will thus have an indirect impact on other patients by limiting the overcrowding of emergency departments. Lastly, given the frequency of suspected pulmonary embolism in emergency departments and the very high usage rate of CT angiographies (80% in a recent observational study [10]), the SPEED&PEPS study should result in substantial savings in terms of the direct cost of diagnostic investigations and the indirect cost of hospitalizations due to waiting times or delayed harmful effects. We expect the cost per serious clinical event avoided in relation to the diagnostic approach to be very favorable compared to current practices.

## Figures and Tables

**Table 1:** 4PEPS or 4-Level Pulmonary Embolism Clinical Probability Score.

Field	Criterion	Points
Demographic data	Age < 50 years old	−2
	Age ≥ 50 years old and <65 years old	−1
	Male	+2
Medical history	Chronic pulmonary disease	−1
	Personal history of VTE	+2
	Hormonal estrogenic treatment	+2
	Immobility within the last 4 weeks ^a^	+2
Signs and symptoms	Heart rate < 80 beats per minute	−1
	Combination of chest pain and acute dyspnea	+1
	Syncope	+2
	Pulse oxygen saturation (SpO2) < 95%	+3
	Calf pain and/or unilateral lower limb edema	+3
Differential diagnosis	PE is the most likely diagnosis	+5
Categories of clinical probability	Very low CP: PE can be ruled out	<0
	Low CP: PE can be ruled out if D-dimer < 1000 µg/L	0 to 5
	Moderate CP: PE can be ruled out if D-dimer < 500 μg/L or <(age × 10) μg/L	6 to 12
	High CP: PE cannot be ruled out without imaging testing	>12

^a^ Surgery (under general anesthesia), lower-limb plaster cast or bedridden > 3 days for acute medical condition within the last 4 weeks.

## Data Availability

Anonymized raw data on individual patients constituting the basis of the analysis, results and conclusions will be available to a third-party auditor and to researchers on reasonable and justified request to the corresponding author (Prof. Pierre-Marie ROY—PMRoy@chu-angers.fr) after approval by the study steering committee.

## Supplementary Materials

The following supporting information can be downloaded at: https://www.mdpi.com/article/10.3390/diagnostics12123101/s1, Figure S1: The PRagmatic-Explanatory Continuum Indicator Summary 2 (PRECIS-2) wheel.
