# Supplementary material for: Diagnostic Strategy for Suspected Pulmonary Embolism in Emergency Departments Based on the 4-Level Pulmonary Embolism Clinical Probability Score: Study Protocol of SPEED&PEPS Trial
Source: Diagnostics (Basel). 2022 Dec 9;12(12):3101. doi: 10.3390/diagnostics12123101 (PMC9777430; doi:10.3390/diagnostics12123101)
Supplement: Supplementary file 1 [file diagnostics-12-03101-s001.zip › diagnostics-2029980-supplementary.pdf]

Supplementary materials

SPEED&PEPS trial - Analysis via the PRECIS-2 grid

SPEED&PEPS study is a pragmatic trial aims to demonstrate the safety and efficacy of an intervention on medical practices. According to the PRECIS-2, it is rated of 42 out of 45 on the scale, with 0 corresponding to a fully explanatory study and 45 to a fully pragmatic study.[24] (Figure 1)

Eligibility: the eligibility endpoints are simple and relate to the target population for which the strategy is intended: patients with suspected PE in the emergency department. The exclusion criteria are limited and justified: patients in shock as well as pregnant women require a specific diagnostic approach and the 4PEPS score has not been evaluated for these specific populations. Similarly, the epidemiology of thromboembolic disease is different in children. In addition, patients with a formal diagnosis before applying the strategy also do not fit the target population. The only exclusion criteria directly related to the study were lack of the ability to follow-up and lack of social security cover. The eligibility endpoint is thus evaluated at 5.

Recruitment: participants are recruited day and night, every day of the week during the study, and by the physicians who normally treat the patients in their usual practice conditions. Patients are recruited at the time they receive treatment in the emergency room, under the usual admission conditions. Patient participation is voluntary and free of charge with no financial or in-kind incentives. The recruitment endpoint is thus evaluated at 5.

Context: the study took place in the emergency departments of university and non-university hospital centers of varying sizes throughout France. The centers involved are representative of the centers where the 4PEPS strategy is likely to be used in France. The context endpoint is evaluated at 5.

Organization: the organization of the study in clusters is perfectly adapted to daily practice. It involves implementing a standardized operational protocol in a care facility. Randomization is only involved at the center level. Real-time data capture using smartphones, tablets, and computers limits the impact of the study on medical practices. The organization endpoint is evaluated at 5.

Adaptability-implementation: the 4PEPS strategy is implemented using the SPEED application, which provides a reminder of care recommendations. The benefit of the application has previously been demonstrated to improve the quality of treatment of suspected PE in the emergency department. Its development with the ability to use it on various mediums will facilitate its implementation. A hard copy of the 4PEPS strategy will also be distributed to stakeholders. Ultimately, it is at the discretion of the clinician to follow the suggested strategy or not. However, the application cannot be integrated with the patient management software of the various participating hospitals. The applicability-implementation endpoint is evaluated at 4.

Adaptability-Adherence: participant adherence to the strategy will not be an issue, as the treatment is coordinated by the physician caring for the patient. Physician adherence to the strategy is supported by the SPEED application. The applicability-adherence endpoint is evaluated at 4.

Follow-up: patients follow-up is performed using medical records and via telephone at the end of the study. National death registers are also consulted if contact cannot be made. As follow-up takes place at the end of the study, with no intermediate follow-up, it is not likely to affect the adherence of study participants. The follow-up endpoint is evaluated at 5.

Primary endpoint: reducing the use of thoracic imaging while maintaining a level of safety at least equivalent to that expected in current practice is a relevant endpoint for both patients and physicians. The safety endpoint represents the net clinical benefit for patients. However, it is an endpoint that combines different elements that are not equally serious, ranging from fatal pulmonary embolism to distal vein thrombosis and major bleeding. The primary endpoint is thus evaluated at 4.

Primary analysis: the primary analysis has two parts with superiority analysis performed on the usage rate of thoracic imaging and a non-inferiority analysis on the safety endpoint compared to the usual treatment. The primary analysis is performed on an intention-to-treat basis with secondary per-protocol analysis of the 4PEPS strategy. The primary endpoint is assessed at 5.

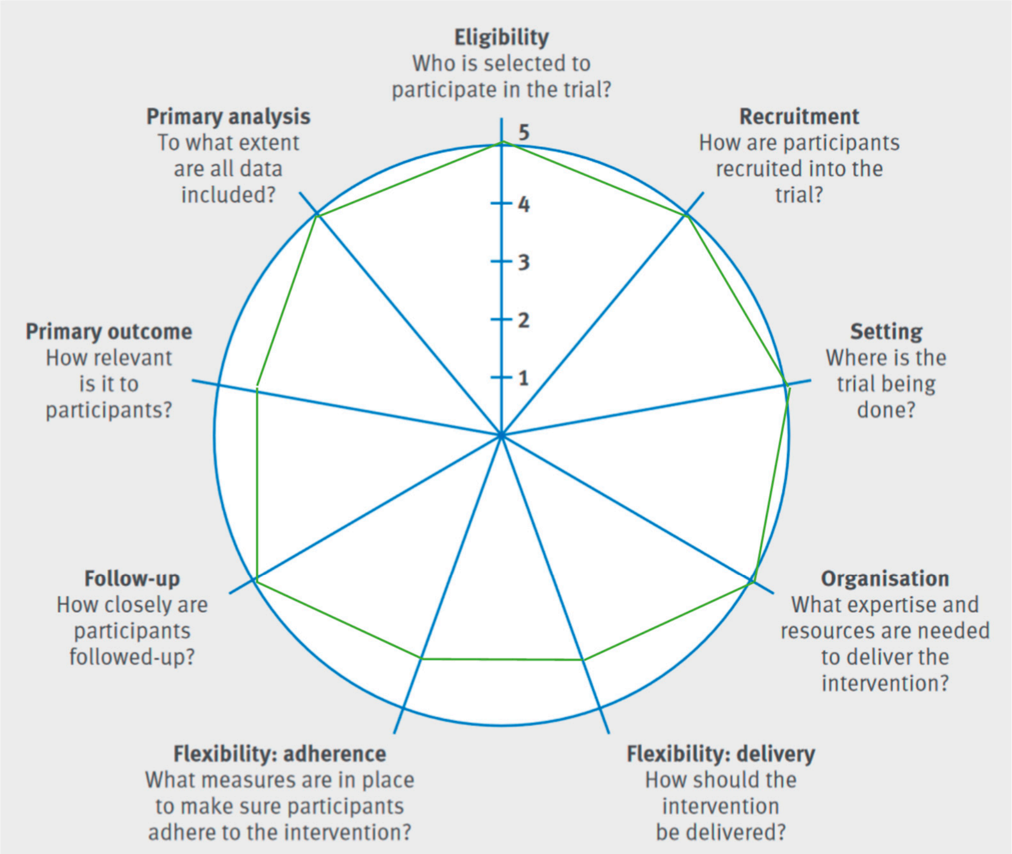

Figure : *The PRagmatic-Explanatory Continuum Indicator Summary 2 (PRECIS-2) wheel.*
